
JOURNAL INFORMATION
==============================
NLM Title Abbreviation: Can Med Educ J
Journal ID: Can Med Educ J
NLM Journal ID: 101560935
Journal ID: CMEJ

Canadian Medical Education Journal

EISSN: 1923-1202
Publisher: University of Saskatchewan

ARTICLE INFORMATION
==============================
PMCID: PMC7162309
DOI: 10.36834/cmej.70084
Article ID: CMEJ-11-e004
Article version: 1
Subjects: Abstracts

Workshop Abstracts

Electronic publication date: 2020 Apr 18
Publication date: 2020 Apr
Volume: 11
Issue: 2
First page: e4
Last page: e30
Copyright: © 2020; 11(2) licensee Synergies Partners
Copyright year: 2020
License: This is an Open Journal Systems article distributed under the terms of the Creative Commons Attribution License (http://creativecommons.org/licenses/by/2.0) which permits unrestricted use, distribution, and reproduction in any medium, provided the original work is properly cited.
License URL: https://creativecommons.org/licenses/by/2.0/

==============================

CMEJ 2020, e4-e30 Available at http://www.cmej.ca

**Sunday, April 19th - 08:00-09:30**

### Workshop Presentation – Block - A

#### WA-1 Successful "in-the-moment" feedback and coaching conversations in the era of CBME and workplace-based assessment

**Rationale/Background**: Feedback is a dynamic and co-constructive interaction in the context of a safe and mutually respectful relationship for the purpose of challenging a learner's (and educator's) ways of thinking, acting or being to support growth (Ajjawi & Regehr, 2019). The R2C2 model for feedback and coaching, with four phases in which supervisors and learners build relationship, explore reactions and reflections, determine content, and coach for change to co-create an action plan, was developed as a model to facilitate such conversations. It was based on theory and research related to self-assessment, cognitive domains, humanism, commitment to change and implementation science. It has been tested and found effective for work with physicians in practice, nurse practitioners, and residents across several countries and disciplines. Recently, the researchers have modified the R2C2 model for use with in-the-moment feedback and coaching that occur in the clinical environment. This workshop will provide participants with an opportunity to explore and practice the R2C2 in-the-moment model (https://medicine.dal.ca/departments/core-units/cpd/faculty-development/R2C2.html) and discuss its applicability within their own context.

**Instructional Methods:** This interactive workshop will draw upon participants' experiences through: 1. Introductions and large group discussion of participant experiences (10 minutes) 2. Presentation and demonstration of the model (15 mins) 3. Small group discussion of the model (10 mins) 4. Coaching practice with case scenarios and debriefing (40 mins) 5. Large group discussion of R2C2 in-the moment coaching and feedback and take-home messages (15 mins)

**Target audience:** Educators at all levels of the education continuum with an interest in feedback and coaching

**Learning Objective:**

1. Share experiences providing "in-the-moment" feedback within clinical settings.
2. Practice applying the R2C2 ITM model to case scenarios.
3. Identify the utility of and barriers to integration of the R2C2 in-the moment (ITM) feedback and coaching model and its application within their work.

### WA-2 Navigating the Evidence Synthesis Landscape: Exploring Fundamentals of common evidence synthesis approaches in medical education

**Rationale/Background:** The growing legitimacy of medical education research is reflected in an unprecedented rise in the number of peer-reviewed publications. This seeming abundance of primary studies has catalyzed the development and/or use of multiple types of evidence syntheses. In choosing a methodology, scholars have been challenged to navigate, identify, and apply both standard and innovative approaches for summarizing research and other forms of evidence. While medical education draws its strength from researchers who embrace diverse epistemologies, methodologies and philosophical approaches, Ellaway et al. note this plurality creates substantial challenges for those attempting to achieve coherence in acts of knowledge synthesis and consequently, their applicability and use. Recent research in medical education has also challenged the application of traditional evidence synthesis approaches, especially focusing on whether and how to employ them well.

**Instructional Methods**: In this workshop, we will explore the nature, purpose, value and discourses of systematic, scoping and realist syntheses. We will also discuss their utility for medical education researchers, as well as how they may be adapted to respond to the unique and changing complexities of our field. Fundamental methodologic characteristics for each synthesis will be compared and contrasted. Further, we will suggest tools that can be used to inform the interpretation and production of these common synthesis approaches. We will use a mix of didactic as well as small group discussion and engagement where participants 'map' question to method using a hands-on approach followed by small group presentation.

**Target audience**: Individuals interested in learning more about the fundamentals of evidence syntheses, those looking to critically appraise and apply findings of common evidence syntheses to their practice (e.g. consumers of synthesis evidence), and those interested in conducting either a scoping, systematic or realist synthesis in future.

**Learning Objective:** As a result of the session, the audience members will be able to: 1. Discuss and discern the nature, purpose, value and role of common evidence synthesis approaches including systematic, scoping and realist syntheses of medical education research; 2. Critically examine assumptions made about different evidence synthesis approaches, including ontological, epistemological, methodological, and conceptual bases, boundaries and innovations related to each; 4. Understand basic methodological considerations of each approach.

### WA-3 Strengthening Health Equity in Undergraduate Medical Education and Continuing Professional Development: Using storytelling and role play to build competencies

**Lloy Wylie** Western University

**Rationale/Background:** Research has demonstrated a range of gaps in addressing the varied health care needs of racialized populations (Wylie et al., 2018, Wylie and McConkey 2019). The aim of this workshop is to demonstrate ways to build competencies among medical students, residents and physicians to advance health equity. There is a need to improve knowledge and practice through targeted educational initiatives that give providers concrete recommendations to improve equity in practice. Such actions include understanding and correcting their own biases, improving their knowledge, and ensuring system-wide supports for culturally safe and appropriate care.

**Instructional Methods:** This workshop uses storytelling, role play and interactive case exercises to demonstrated strategies for building competencies in providing culturally safe, quality health care for Indigenous people, immigrants and refugees. This workshop will provide experiential learning opportunities that demonstrate strategies that build concrete skills. In addition, the workshop will support participants to develop theatre-based training activities in their own health service settings. The presenters will illustrate a range of challenges facing Indigenous people, refugees and immigrant, both within the health care system and more broad social determinants of health. Narratives drawing on examples from health care experiences that demonstrate challenges will be presented. Participants will work in teams to explore the issues in the cases, reflecting on determinants of health through role play exercises. The workshop facilitators will guide a reflection on the process and explore ways participants can apply this approach to their own settings.

**Target audience:** This workshop is oriented to health care providers, health care educators, and others involved in training health professionals.

**Learning Objective:**

1. To identify the range of challenges that undermine health equity.
2. To demonstrate evidence-based exercises that can improve health care providers' attitudes, knowledge and skills in promoting culturally safe changes in health care delivery
3. To support participants in developing educational and workplace strategies applicable to their own setting

### WA-4 Leveraging Data Visualization and Data Storytelling in Programmatic Assessment and Program Evaluation

**Rationale/Background:** Storytelling is a powerful tool in transforming the way people remember and respond to information. Research in cognitive sciences indicate that humans have been telling stories for thousands of years and human brains are wired to process and respond to them. Both Programmatic Assessment and Program Evaluation in medical education involve collecting large volumes of data and making informed decisions using that data. The use of online exam platforms and survey tools enables us to collect the data, but effective presentation and communication of assessment and evaluation data is still evolving. In the past 3 years, our office explored the use of data visualization and storytelling to support data exploration and analysis, high-stakes decision making, exam bank quality auditing, and identifying areas of improvements as a part of our overall Program Evaluation Model.

**Instructional Methods:** We will begin by presenting theories and best practices in data visualization and data storytelling. A brief presentation of real examples of data visualization from our office will follow. We will then divide participants of different levels of expertise into small working groups to engage in two visualization and storytelling design activities. There will be a Q&A session at the end followed by a brief wrap-up.

**Target audience:** Administrative leaders, department heads, education scientists, data practitioners and staff who are involved in medical school assessment and evaluation areas.

**Learning Objective:**

1. Identify data visualization and storytelling opportunities in student assessment and program evaluation.
2. Learn how to present quantitative information effectively
3. Develop confidence in effectively engaging and persuading your audience with storytelling

### WA-5 Getting your ducks in a line: enhancing research congruence

**Rationale/Background:** Axiology, ontology, epistemology, theory, methodology, methods: intimidating words regardless of experience. As researchers, we naturally gravitate towards the familiar, preferring to discuss research methods - "I'm doing a focus group study"- than grapple with these intimidating underlying concepts. Failing to attend to these key elements, and their alignment, however, will cause problems throughout all stages of research

- not least when a researcher pursues publication or defends their thesis. Congruent research is a key tenet of rigour. Participants will feel supported to apply their learning to their own research contexts and ultimately engage with more rigorous work.

**Instructional Methods:** This workshop will support participants to explore these fundamentals of rigorous research through practical and memorable tasks. Working primarily in small groups, interspersed with some focused mini-lectures, participants will configure 'worked examples' in congruent ways and consider how improved congruence might enhance their own research projects.

**Target audience:** Anyone interested in enhancing the rigour of their research will have a valuable contribution to make. Our activities, however, will be of particular benefit to more novice researchers or those at early stages of project conceptualisation and planning.

**Learning Objective:**

1. Explore why research congruence matters; and matters from the outset
2. Identify key distinctions between concepts outlined in workshop background •
3. Develop key action points as to how they will translate this learning into their own research practice.

### WA-6 Price of a dream: Strategies to reduce medical school application costs for students from low socioeconomic status backgrounds

**Rationale/Background:** Canadian medical schools are responsible for training a physician body that is representative of the population they serve, and have a mandate for diversity and inclusion in their student body (1). Physicians are more likely to serve a population that reflects their own background, and doctors who share a background with their patients are perceived by patients to provide better care. However, students from low SES backgrounds are severely underrepresented in the medical student body (2), with application costs significantly limiting their access to medical school (3).

**Instructional Methods:** After introductions and eliciting participants' interests, we will introduce a key focus area of the University of Toronto (UofT) MD admissions and outreach team - educating applicants about the financial cost of pursuing a career in medicine. We will describe data gathered on the general (cost) elements of medical school admissions process and total costs per applicant type, and share the UofT MD Program experience in implementing key strategies to educate and support applicants (20 min). Participants will then work through scenarios in small group discussion with large group debriefing to identify and share resources, personal experiences, and collaborative methods for reducing application-related costs (50 min). Next steps and potential strategies will be highlighted to conclude the session (20 min).

**Target audience:** Admissions and registrarial staff working with premedical students and admissions committees members.

**Learning Objective:** In this presentation we will highlight the various direct and indirect costs associated with the admissions process for Canadian prospective medical students and provide administrative leaders with advice on how to implement strategies to reduce these costs.

### WA-7 Reviewing in Medical Education: The CMEJ experience

**Marcel D'Eon** University of Saskatchewan

**Rationale/Background:** Reviewing papers is an important contribution to the medical education community and an excellent learning experience. Reviewing is a lonely activity with little feedback or opportunity to collaborate. With support from the Editor and select Associate Editors (AEs) participants will learn about the peer review and decision making process at the CMEJ. They will work independently and in small groups to review one or two actual original submissions that were eventually published in the CMEJ.

**Instructional Methods:** Introductions, presentation of the peer review process at the CMEJ as well as the main reasons for revisions and/or decline decisions. Participants will choose one of two or three original submissions to begin to review individually; Editor and AEs will circulate and provide assistance as needed. Participants will form pairs and/or small groups to compare their observations and critiques. Editor and AEs will monitor each group and provide assistance as needed. We will end with a Q&A period and a general wrap-up.

**Target audience:** All faculty, staff, residents, and students interested in strengthening their skills in critical appraisal and advancing their contribution to medical education in Canada through reviewing.

**Learning Objective:** Apply (better apply) 2-3 skills in reviewing papers in medical education Navigate the CMEJ website to review submissions Anticipate tasks, features, and reminders associated with the CMEJ reviewing process

### WB-2 Intégrer des patients partenaires: comprendre les enjeux terrains

**Rationale/Background:** Évaluer, superviser, monter une activité d'enseignement, faire de la recherche, améliorer les soins, gérer une clinique ; de plus en plus d'initiatives vivent à impliquer des patients partenaires dans ces différents contextes partout à travers le Canada. À partir des résultats d'une étude qualitative réalisée dans le cadre d'une maîtrise en pédagogie médicale, nous proposons un atelier pratique pour permettre aux participants d'analyser et adresser les enjeux potentiels lorsque cliniciens et patients travaillent ensemble à l'extérieur d'un simple cadre thérapeutique.

**Instructional Methods:** Par le biais de mises en situation, et de discussions interactives en groupe, les participants pourront analyser des situations et des enjeux pratico-pratiques rencontrées sur le terrain. À l'aide d'une grille d'analyse issue de la recherche, ils seront appelés à réfléchir sur le changement que soulève l'arrivée du partenariat avec les patients sur leur pratique comme enseignant ou comme cliniciens.

**Target audience:** Tous ceux appelés ou intéressés à travailler avec des patients partenaires

**Learning Objective:**

- Analyser les enjeux potentiels lorsque cliniciens et patients travaillent en partenariat
- Réfléchir à des pistes pour répondre aux enjeux terrains
- Comprendre comment le partenariat patient peut toucher l'identité des professionnels de la santé

### WB-3 Peer observation of teaching: the next step in Faculty Development

**Rationale/Background:** It is recognized that being a good clinician is not enough to be a good teacher. Several faculties have a faculty development program. However, clinical teachers remain poorly observed and receive little feedback on their teaching skills. The expansion and decentralization of several programs has increased the need for faculty training and sometimes led to distance constraints. A method of peer observation of teaching (POT) with feedback can be helpful in addressing both of these issues. This practice can be fostered by the use of a simple, user-friendly tool that must still include the essential attributes of an effective teaching clinician as already described by several authors and in the Fundamental Teaching Activities Framework of the College of Family Physicians. The tools found in the literature are often long and exhaustive, making them difficult to use in a busy practice. This is what motivated the author to develop the tool that will be presented and used during this workshop.

**Instructional Methods:** • Brief formal presentation: context, presentation of the tool • Practice the method using the tool: role-playing games in trio • Debriefing following role-playing • Group discussion: examples of practical applications • Key messages

**Target audience:** Clinical supervisors of all level and faculty developers

**Learning Objective:** During this workshop, participants will be able to: • Familiarize themselves with the concept of POT. • Practice POT and giving feedback using a tool that facilitates feedback between colleagues. • Discuss its practical application for local and continuous faculty development.

### WB-4 Professionalism: Developing a Vision for Evidence Informed Curriculum

**Rationale/Background:** Unprofessional behavior amongst learners and health professionals continues to be problematic. Teaching, assessing, and remediating professionalism is challenging, due to its multifaceted nature, the influence of context, and lack of a clear definition. This can lead to a "shotgun" approach to the professionalism curriculum where multiple methodologies are employed in the hope that "something will work" to capture the essence of professionalism. While this approach may have merits, a more targeted and evidence informed approach is needed. In this workshop, four conceptualizations of professionalism (competence, behaviors, values and identity) will be explored with a focus on designing curricular approaches to address each of these. Participants will explore and co-create curricular approaches which are evidence informed and are clearly linked to specific professionalism outcomes.

**Instructional Methods:** Following a brief overview of how professionalism is conceptualized in the literature, participants will break into small groups to discuss and strategize how to teach, assess and remediate professionalism utilizing one of the four conceptualizations. Groups will report back for large group discussion and debate, and end with a case study to apply the concepts to real life scenarios.

**Target audience:** All health professionals involved in the teaching of professionalism, in both academic and clinical settings. Content will be applicable to both novice and experienced educators.

**Learning Objective:** By the end of the workshop, participants will able to align teaching, assessment, and remediation methods with four key elements of professionalism to develop a more targeted and evidence informed professionalism curriculum.

### WB-6 Making workplace-based assessment work; recognizing opportunities and limitations

**Rationale/Background:** The number of tools for workplace-based assessment (WBA) in under- and postgraduate medical education have steadily increased over the past decade and with it conceptual models for their use have evolved. Evaluation studies, however, indicate that WBA in clinical practice does not always work as well as in they should in theory. Assessment procedures can become tickbox excercises and assessment for learning degrades to (merely) being perceived as summative assessment. This workshop will focus on these challenges by asking what practitioners and educationalist can do to make WBA work for learning.

**Instructional Methods:** The workshop will use the following approaches: small group learning in a round robin design and moderated plenary discussion. We will build on participants' experiences with and knowledge of WBA. After a brief plenary introduction by the moderators, a round robin design will be used to enable small group discussions about four different WBA related topics during the first workshop hour. One topic concerns the tension inherent in WBA related to assessment for vs. of learning. One deals with the question what matters in assessment, for whom and why. The final two topics address do's and don'ts for trainees and preceptors. An interactive plenary discussion will allow synthetisation of outcomes and lead to key findings.

**Target audience:** We expect students, residents, faculty and educationalists to benefit from insights in how WBA can work in clinical practice.

**Learning Objective:** Participants will be able to: - better understand how inherent tensions in assessment at the workplace affect WBA - understand under what circumstances WBA can optimally support learning in practice, - know what role individual trainees and preceptors can play in making WBA work for them.

### WB-7 Applying the 3-P Model to Plan and Evaluate your Interprofessional Education Activity

**Rationale/Background:** Interprofessional Education (IPE) positive outcomes are being recognized, with governments and educators calling for IPE. IPE provides an opportunity for colleagues to work together in teams, enabling development of knowledge, skills and attitudes to practice collaboratively in the workplace, thereby enhancing service delivery. The 3P model has been previously proposed as an effective model for describing and analyzing IPE learning activities. It uses narrative descriptions of presage, process and product factors of the educational experiences, and focuses on the complex and dynamic relationships between the different factors.

**Instructional Methods:** Innovative, learner-centred and co-creative instructional approach using a restaurant conceptual framework ("MedEd à la carte"): 1. Facilitators do a "Needs Assessment on-the-fly" and assign seats according to learner characteristics (a. Phase in the educational cycle: planning vs evaluation; b. IPE Expertise: 101 vs 202; c. Phase in the education continuum: Undergrad, Postgrad, CPD); 2. Menu organized with 3P framework: Presage, Process and Product; 3. Participants at each table select topics to be discussed; 4. Facilitators select from pre-set provocative questions related to the topic and table's needs; 5. Questions are served for participants (small group discussion); 6. Whole group discussions take place at the end of each "course"

**Target audience:** Clinician teachers and clinician educators from any level of the education continuum interested in, planning, implementing, analysing or evaluating an interprofessional education activity

**Learning Objective:** Apply the Presage, Process and Product model to the planning and evaluation of their IPE activities; Discuss potential drivers, barriers and approaches associated with positive IPE outcomes.

Monday, April 20th - 10:00-11:30

## Workshop Presentation – Block - C

### WC-2 Practical Strategies for Meaningful Allyship in Indigenous Health and Beyond

**Rationale/Background:** Well-intentioned efforts towards healthcare equity and diversity may backfire and erode trust and allyship. Existing frameworks and instructional strategies have shortcomings or limitations. Allyship is a concept that may have relevance to advance efforts for healthcare equity into the future, yet is under-explored in healthcare and health professions education. This session will provide an introduction to the concept of allyship, how it is distinct and/or complementary to other concepts (e.g. cultural humility, anti-oppressive practice, etc.), while co-creating practical strategies for participants to build and practice allyship in their organizations.

**Instructional Methods:** • Icebreaker/reflection exercise through think-pair-share activities • Brief didactic with handout for introduction to terms and concepts • Storytelling and co-creation • Small group case discussion with large group debrief

**Target audience:** All are invited to participate and join the dialogue

**Learning Objective:**

1. Define the concept of allyship and how it is distinct or complementary to other related concepts in health professions education
2. Provide examples of how to enact allyship as it relates to indigenous health
3. Describe and practice strategies to build and enact allyship within own organization(s)

### WC-3 Teaching how to deal with emotions during difficult conversations: Opportunities and challenges

**Rationale/Background:** High-quality communication in healthcare results in greater patient and family satisfaction. Care models like patient- or person-centered care (1) and shared decision-making(2) have been increasingly promoted to improve physician communication. These models focus both on information sharing and emotional support. Yet, teaching how to provide emotional support during communication training presents unique opportunities and challenges(3).

**Instructional Methods:** The workshop will be divided in three parts. 1- Participants will first engage in video review and role playing of difficult conversations to help them reflect on a) their own personal emotional responses to difficult conversations, and b) communication strategies used to address patients and families' emotions. 2- We will then propose a communication toolkit useful for emotional conversations that will be discussed and practiced with participants. 3- Finally, participants will be asked to design, in small groups, a communication training outline focused on emotional support using one of three proposed educational strategies.

**Target audience:** Medical educators and teachers involved in the design or delivery of communication skill training and assessment.

**Learning Objective:** By the end of the workshop, participants will be able to:

1. Explain the importance of emotional awareness and support during difficult conversations with patients and families;

2. Demonstrate simple communication skills helpful for emotional support during difficult conversations with patients and families;
3. Compare different educational strategies to help trainees to support emotionally patients and families.

### WC-4 Discovering Cultural Humility in Healthcare Education

**Rationale/Background:** Hospitals in large urban centres like Toronto have witnessed a vast increase in the number of international learners, patients and providers that bring different lived experiences and ideas of culture. Our organizations, by default, are shifting towards becoming increasingly international in their makeup. Arguably however, there seems to be a disconnect between the structures we work in and the populations they serve. The workshop explores the notions of cultural humility and diversity and how it can enhance our teaching and learning practices in health professions education. By using their lived experiences as a personal lens, and by practicing through role plays, participants will be encouraged to challenge their worldviews, mental models, and current systems. Participants will also reflect on the western philosophies of learning they have been socialized to, and espouse, in health care settings.

**Instructional Methods:** Workshop style with group discussions, role play and case studies

**Target audience:** Healthcare providers, educators, administrators, leaders

**Learning Objective:**

1. Explore the concept of cultural humility and how it can enhance our teaching and learning practices
2. Explore the intersection of culture with teaching & learning and the impact has on our lens as educators, providers and learners
3. Challenge current ways of thinking, systems and philosophies.

### WC-5 Side by Side : A peer support program for medical students at the University of Ottawa

**Rationale/Background:** Depression affects nearly 1/3 of medical students worldwide. Among medical students who screened positive for depression, only 15% sought treatment. In an age-matched sample of the general population, psychological distress was shown to be lower in students entering a medical program. Mental health then deteriorates during medical school. The importance of well-being among medical students includes: enhancement of reflective practice, communication skills, empathy and patient care. Depressed residents and doctors are likely to make six times more medication errors than colleagues who aren't depressed. Very few peer-led programs have been assessed in a medical student population. Respondents from peer-support and mindfulness program "favoured support services that are non-stigmatizing, accessible and confidential, preferably provided by peers with medical training like themselves." There is evidence that peer-support or "peer-led" programs can improve help seeking behaviors and suicide perceptions, increase social integration, and reduce anxiety scores, among students in secondary school and university settings.

**Instructional Methods:** A student-led peer support program, such as Side by Side, is a valuable addition to the current programs and services offered at our faculty. The goals of the programs are to: improve help-seeking behaviors; reduce stigma; Provide non-judgmental, accessible and confidential peer support from students who have undergone professional training; approach peers who are at risk of experiencing distress based on behavioral changes; facilitate students' pathway to proper resources and professional services. This is an interactive workshop representing the implementation of such a program and the training presented to peer support students. The instructional methods used during this workshop are small and large group scenarios and discussions as well as hands on techniques to facilitate interactions. Participants will be provided with a training manual with questionnaires and reflection tools aimed at presenting the following main topics: 1. Peer Support 101 2. Communication & Basic Counselling 3. Responsibilities, Limits & Resources 4. Crises & Suicide 5. Common Scenarios & Practice 6. Self-Care

**Target audience:** This workshop targets students, residents, student affairs, faculty wellness, counselors and any faculty member with interests in wellness and mental health.

**Learning Objective:** At the end of this workshop, the participant will:

1. Recognize the need for prevention, identification, and treatment of mental illness early in medical training;
2. Identify factors that contribute to poor help-seeking behaviors in medical students;
3. Describe the implementation of a peer support program in medical school including planning, recruitment, training, and evaluation.

### WC-6 Deriving Logic in Program Evaluation: Application of Logic Model Frameworks in Health Professions Education.

**Rationale/Background:** Determining the effectiveness and value of educational programs is paramount to the success of Health Professions Education (HPE). However, making sense of program functioning is complex, due to the myriad factors that interact to generate program outcomes [1]. With the introduction of Competency Based Medical Education (CBME) - an outcomes-based approach [2] - it is important to evaluate outcomes while offering an understanding of how outcomes are generated. This workshop introduces attendees to the use of program logic models - which are an evaluation framework [3] that would help them understand the linkages between program activities, processes, and program outcomes.

**Instructional Methods:** We will begin with a didactic overview of the Logic Model (LM) framework as a way to structure the evaluation of educational programs. Through structured small group exercises, participants will work on refining their understanding of LMs and develop evaluation plans for their programs. Workshop faculty will work closely with attendees to provide personalized assistance progress during the session. Participants will be asked to present their work back to the group for facilitated feedback, including a Q&A section to clarify any questions that might have resulted after the activity.

**Target audience:** This workshop is intended for early career educators, researchers, students, and administrators that are interested in understanding the fundamentals of high-quality evaluation practice. No previous knowledge around program evaluation is required.

**Learning Objective:** By the end of this workshop, participants will be able to use logic models to facilitate evaluation planning for complex educational interventions.

### WC-7 Capacity Building for Case-based Quality and Safety Education using QuaCC: the Quality Care Curriculum

**Rationale/Background:** Quality Improvement and Patient Safety (QIPS) teaching is becoming widely accepted as a vital aspect of modern medical education (1). Despite this, there exist several barriers to its effective implementation in the clinical context, including limited skills, resources, and opportunities for applied and interactive learning (2). Many institutions rely on a combination of didactic lectures with mandatory QIPS projects for residents or medical students (3). We have designed an innovative, longitudinal, case-based QIPS curriculum, which addresses learning needs across undergraduate, post-graduate, sub-specialty, and faculty development levels. Each session focuses on the application of a quality, safety or leadership concept to a real case, which is identified and presented by the learners, followed by a facilitated, clinically relevant discussion.

**Instructional Methods:** The workshop will build capacity to facilitate case-based QIPS education sessions through the use of small group activities. Groups will be provided with preparatory materials and be coached through delivering and participating in fun, interactive, and adaptable sample sessions. An approach to the implementation and evaluation of such a curriculum will be explored.

**Target audience:** Clinical educators (Faculty, Fellows, Senior Residents) with an interest in QIPS.

**Learning Objective:** By the end of this session, participants will be able to

1. Develop a curriculum for case-based learning of QIPS materials
2. Deliver QIPS rounds to engage learners at all levels
3. Evaluate the success of their case-based QIPS curriculum in a clinical context.

Monday, April 20th - 13:00-14:30

## Workshop Presentation – Block - D

### WD-1 Assessment of the Individual within a Team Context

**Rationale/Background:** Physicians work as part of large teams and patient outcomes are rarely related to the competence of an individual physician, but rather are related to the competence of the team. Despite this, assessments within CBME frameworks are based on the competence of the individual trainee. In this workshop, the presenters will discuss circumstances in which trainee's actions, decisions, and performance are not independent, setting the stage for the need for innovative assessment strategies that examine individual competence within a team context. Presenters will discuss the challenges of assessing for collective, team performance in the complex clinical environment and then share their experience with two strategies for assessing individuals whose performance is dependent on other team members. One of these assessment strategies is based on studies in educational measurement and provides an approach for assessing collaboration to capture aspects of independent and non-independent performance along a spectrum by describing individual relations with different team members and their contributions to outcomes. The second strategy is based on Multi Source Feedback (MSF) and uses team members' assessment of the individual trainee on a particular case to capture trainee's and team members' contributions. These assessment strategies attempt to determine the contribution of the trainee to the final clinical outcome rather than attributing the clinical outcome to the individual trainee. In the last part of this workshop, the authors explore the validity of these assessment strategies and whether qualitative assessment rather than quantitative might be better suited to capture the "Collective Competence"

**Instructional Methods:** Large group discussion on the need for new assessment strategies Mini lecture with small group case study and demonstration of strategies Mini lecture and small group discussion on validity of these assessment strategies

**Target audience:** Educators whose responsibility is to assess trainees

**Learning Objective:** Discuss the need for innovative assessments that look at the individual trainee within a team context Describe two strategies to assess individual and team contribution to the final performance and clinical outcome Explore the validity of these two assessment strategies

### WD-7 Coping with Imposter Syndrome in Medical Training

**Rationale/Background:** As wellness consultants working with postgraduate medical trainees at University of Toronto and McGill University, we often help residents and clinical fellows struggling with Imposter Syndrome and perfectionistic anxiety. The culture of postgraduate medical training can inadvertently foster and/or exacerbate feelings of self-doubt and inadequacy. Trying to learn while expecting oneself to adhere to unrealistic perfectionistic standards can hinder both learning and clinical performance. Shifts in one's mindset and the broader culture of learning environments are possible and can lead to safer, more flexible environments in which to learn.

**Instructional Methods:** We will review relevant theories and research with respect to Imposter Syndrome, perfectionism, mindsets, decision making, and psychological safety in terms of their applicability to medical training. We will aim to provide tools for trainees to manage their own distress and enhance their learning and clinical performance, and help trainees and educators gain knowledge about practical and achievable changes within the medical training environment to foster safer spaces to learn and work. We will incorporate interactive exercises including self-assessment, reflection, small and large group discussions, and experiential use of strategies to manage discomfort.

**Target audience:** Postgraduate medical trainees and educators

**Learning Objective:** Participants will learn to:

- Recognize thoughts, behaviours, and emotions related to Imposter Syndrome
- Use skills to manage perfectionistic anxiety and self-doubt
- Recognize roles they may play in perpetuating a culture of unrealistic standards
- Identify ways to create safer, more supportive learning environments.

### WD-6 Raising critical consciousness: introducing social justice to generalist clinical teaching

**Rationale/Background:** Family doctors and other generalists, embedded in communities, are often acutely aware of social determinants of health. Their way of knowing medicine encompasses a commitment to humanism, relational care, and health advocacy. For these professionals, core skills of managing complexity and uncertainty are mediated by a social justice agenda. However, despite being fundamental to generalist identity and practice, these nuances are rarely acknowledged within formal curricula.1 In this workshop, we encourage clinical educators to make explicit concepts of critical consciousness, and to develop meaningful strategies for translating them. We draw on Freire's critical pedagogy2 to help educators develop their teaching of health advocacy and critically conscious attitudes.

**Instructional Methods:** In the words of Leonard Cohen, we will seek 'the crack in everything; that's how the light gets in'. Working primarily in small groups, interspersed with a few focused mini-lectures, participants will identify challenges and opportunities within our own contexts, to allow critical consciousness to become embedded within teaching, rather than a 'tick box'. We will explore possible educational practices that, adapted and applied to context, can foster students' commitment to address social issues in healthcare.

**Target audience:** Clinical teachers and educators, curriculum designers, and anyone interested in advancing social justice issues in medicine.

**Learning Objective:** In this workshop participants will:

- Identify aspects of their own critical consciousness as educators,
- Explore various ways to be an effective preceptor of critical consciousness,
- Develop practical educational strategies for use in their own teaching contexts.

### WD-5 "Tuning in to Collaborative Healthcare: a new interprofessional education experience in the performing arts."

**Rationale/Background:** While Interprofessional Education (IPE) is a relatively new buzzword in the medical education community, disciplines in the arts and humanities have long been exploring the theory and real-life practice of interprofessional collaboration.[1] In a new and innovative IPE mini-course at Dalhousie Medical School, we draw on professional classical music ensembles in the community to teach communication, team functioning and conflict resolution to healthcare students. The first iteration of this course took place in March 2019 and paired a string quartet with healthcare students in various disciplines.[2] After an incredibly successful debut, student's final reflections described the unique value gained from being fully immersed inside a musical ensemble to observe and engage with professional collaborators as a means to learn what successful collaboration looks like, sounds like, and feels like.

**Instructional Methods:** Methods are modeled after traditional practices in classical music ensembles. Workshop will consist of musical demonstrations by co-presenters (piano & violin) interspersed with guided interactive activities. Attendees will be divided into groups to participate in improvisation exercises around themes of communication, drawing on ideas/teaching points presented in the collaborative musical demonstrations. Emphasis will be placed on exploring conflict resolution through simulated group communication.

**Target audience:** Medical and health educators, particularly those interested in the complex interpersonal communication created in interprofessional health care teams.

**Learning Objective:** We hope to generate interest in medical humanities education opportunities, and leave participants with an understanding that, "Although medicine is inarguably a learned profession, clinical practice is above all a matter of performance."[3]

### WD-4 Is conducting a BEME review worth it? Advantages, challenges, and practical tips

**Rationale/Background:** Excellence in health sciences education requires using and synthesizing credible evidence. The BEME (Best Evidence in Medical Education) Collaboration plays an important role in championing this goal by supporting and publishing systematic, scoping, and realist syntheses. As well, with approximately 60 published BEME reviews and many more in progress, there are still numerous questions that merit further investigation. As members of BEME International Collaborating Centers, we are eager to encourage Canadian scholars to be part of this international trend. During this workshop, we will discuss some of the advantages of undertaking BEME reviews and introduce participants to the process of conducting a BEME review. In addition, based on our experiences with a number of BEME reviews, we will share lessons learned in developing protocols and conducting knowledge syntheses and provide participants with some practical tips and recommendations to overcome commonly perceived challenges.

**Instructional Methods:** Brief interactive large group presentations, individual exercises, and small group discussions

**Target audience:** Healthcare professionals, faculty members, and students who are interested in conducting a knowledge synthesis in medical education

**Learning Objective:** By the end of this workshop, participants will be able to:

- Describe the benefits and challenges of conducting a BEME review
- Identify the various steps required in conducting a BEME review
- Explain the key components of a BEME protocol
- Recognize relevant topics and research questions for review

### WD-3 Educating future educators: Designing and implementing elective courses in medical and health sciences education

**Rationale/Background:** At some point in their careers as health professionals, most clinicians are expected to take on the role of educator, facilitating learning amongst students, patients or other target groups. During the last decade or so, several schools and universities have started to offer elective courses in health professions education with the overall aim of helping students gain insight into how learning can be facilitated on a variety of levels. Most health professions training programs, however, do not have key curricular structures in place to adequately prepare learners for their roles as teachers, supervisors and assessors.

**Instructional Methods:** We will begin by sharing our own varied experiences developing and implementing elective courses in medical education at McGill (Montreal, Canada) and University of Bergen (Norway). We will also share some of our preliminary findings and outcomes, including evaluation results and learning resources produced by students as part of their medical education elective training. The lion's share of the workshop will be dedicated to engaging participants in discussions of how they might design and implement a health professions education elective within their own institution. Through small group activities, participants will be encouraged to formulate suitable learning outcomes, teaching and learning activities and assessment strategies that will serve as first steps toward designing their own unique courses. Ideas will be shared and discussed between the groups. We will complete the workshop by discussing some of the pitfalls, challenges and obstacles we have encountered during our own experiences creating new medical education electives.

**Target audience:** Introductory

**Learning Objective:** By the end of this workshop, participants should be able to:

1) Outline reasons why teaching students in the health professions the foundational principles in education is essential for their future careers as clinicians and medical educators
2) Describe different ways of designing health professions education electives
3) Propose concrete ways they might design and implement elective courses in health professions education in their own settings
4) Recognize the pitfalls, challenges and obstacles of health professions education elective design

### WD-2 Medical student mistreatment 2020: The Calgary Experience of Pushing for Culture Shift Using Faculty Advocates. A Case-based Workshop

**Rationale/Background:** Learners at Canadian medical schools experience mistreatment (1,2). Evidence in the literature is lacking on how to best address issues of student mistreatment or help shift the culture to prevent its occurrence. In 2016 the University of Calgary created the position of Faculty Advocate Against Mistreatment (FAAM), currently occupied by 3 faculty members. An easily accessible web portal enables students to contact the FAAMs directly. Since inception, the FAAMs have been contacted over 100 times with a variety of concerns. This number continues to increase annually and outcomes range from student validation to regulatory body complaints.

**Instructional Methods:** This workshop will utilize an assortment of situations to learn from the Calgary experience. Cases range from student-student conflict to faculty-student interactions with themes of discrimination, physical contact and sexual advances. Through small group case discussions facilitated by FAAMs and students, we will explore the processes used to help students navigate the system as well as the potential outcomes.

**Target audience:** We invite faculty, residents and students from all health professions to learn together in this interactive workshop.

**Learning Objective:** Practice strategies to navigate concerns of student mistreatment in medical education.

### WC-1 Building Gryffindor: Revitalizing the Clinical Education Environment for Engagement & Learning

**Rationale/Background:** The workshop provides participants the opportunity to design and prototype the optimal clinical learning environment that supports patients, caregivers, preceptors, and residents/students. Engaging people in the process of design encourages collaboration, networking, and innovation.

**Instructional Methods:** Experiential learning

**Target Audience:** Clinical educators, residents, and administrators.

**Learning Objective**:

1. Participants will learn about the emerging trends of innovative space and its influence on the clinical learning paradigm.
2. Participants will be able to describe the role of the physical environment in learning, innovation, and creativity.
3. 3. Participants will be able to identify factors in learning spaces that foster interdisciplinary practice.

### WE-1 Serious about Gaming: A workshop for budding serious board game developers

**Rationale/Background:** From hand-washing to resource stewardship, serious games can be used to highlight important teaching points and to provide insights that cannot be achieved with other teaching modalities. GridlockED is a board game developed to help medical trainees better understand the workings of the emergency department and to provide a low-stakes method to practice managing patients in complex clinical environments. TriagED is a game that simulates field triage for paramedics. DischargED is a multiplayer game about managing a ward of patients and effectively arranging for safe discharges for all of them. Using our games as case studies, we aim to teach educators the value of serious games in allowing players to repetitively experiment and experience scenarios in an abstract way.

**Instructional Methods:** Attendees will first be divided into groups to play one of three games (GridlockED, TriagED, or DischargED). A didactic component will follow to provide an overview of the process utilized to construct and test GridlockED as well as some insights into our other game development processes. Finally, the workshop will conclude with a question and answer period outlining the post-development phase and game production.

**Target audience:** This session is designed for attendees who would like to use a serious game to create a novel learning experience for trainees.

**Learning Objective:** Upon completion of this session, participants will be able to:

1) Play above games and begin thinking about opportunities in their own disciplines to design a serious game
2) Compare how a serious game and other classroom-based strategies differ in their ability to teach certain topics (e.g. collaborating with other healthcare professionals)
3) Articulate the steps to creating a serious game.

### WE-8 Teaching the Patient's Medical Home - from policy to practice

**Rationale/Background:** The Patient's Medical Home (PMH) model is a made-in-Canada vision that embraces values of equity, fairness, and access to care for all.1 It is the foundation of health care policy at a federal and provincial level, informing development of health care services in family medicine and generalist specialty services. Despite its influence at a policy level, reports of curriculum interventions at an undergraduate and postgraduate level promoting PMH are relatively absent from Canadian literature. The aim of this workshop is to generate a community of practice interested in developing curriculum interventions and evaluation on PMH by sharing examples of initiatives and identifying next steps for action.

**Instructional Methods:** Following a brief introduction to PMH, workshop facilitators will describe how two Canadian medical schools are implementing PMH curricular initiatives. Participants will then be invited to complete a 'report card'2 to reflect individually on PMH teaching at their institutions. This will be followed by small group discussion to identify areas of commonality and innovation. Next, as a large group, participants will discuss facilitators and barriers to promoting PMH, particularly across specialties and the educational continuum. Finally, participants will identify specific priorities to advance PMH teaching in their context.

**Target audience:** Open to medical educators from all specialties and disciplines, at under- and post-graduate level, including medical students.

**Learning Objective:** Define core concepts of the Patient's Medical Home Conduct a needs assessment on Patient's Medical Home in their curriculum Apply strategies to teach Patient's Medical Home model in their setting.

### WE-9 Educating for Critical Consciousness in the Clinical Teaching Environment

**Rationale/Background:** Medical educators aiming to foster the development of socially responsible trainees may look to transformative education and critical pedagogy approaches. Specifically, critical consciousness endeavours to empower learners to challenge the status quo by illuminating power imbalances and socially embedded inequities. However, there is limited guidance from the literature on how educating for critical consciousness is enacted in a clinical teaching environment. We conducted a qualitative study to understand what critical consciousness looked like in everyday clinical teaching, and the conditions that enabled this practice. We interviewed trans/non-binary patients and academic family health team clinicians to elicit stories of re-humanizing practices and transformative educational moments. We found that educating for critical consciousness in the clinical environment was layered and complex. Our findings provide a framework to guide faculty development efforts focused on integrating critical pedagogy into medical education, orienting medical students toward social action.

**Instructional Methods:** This workshop will review the findings of our study within our specific context of trans/non-binary patients in a Toronto family health team environment. We will then move beyond this context to discuss how critical pedagogy may be enacted in various settings with patients who face disadvantage. This workshop will be highly interactive and employ teaching strategies from the transformative paradigm of education, including critical reflection and dialogue.

**Target audience:** Anyone involved in health professions education

**Learning Objective:** To engage in dialogue about the role of critical pedagogy in the clinical teaching environment and explore ways of navigating the challenges encountered when creating space for this form of education.

### WE-2 "Welcome to medical school!": Designing an inclusive medical school orientation for medical students

**Rationale/Background:** Medical school orientation is a complex period of transition that is understudied. We conducted a national qualitative study exploring the medical school orientation experiences of first year medical students. Our study revealed a multitude of tensions experienced by incoming medical students, namely, tensions with themes surrounding diversity and inclusion, professional identity development, and socialization. The goal of this workshop is to invite a broad range of stakeholders to actively participate in a discussion that builds on our research findings, with the ultimate goal of building consensus for tangible suggestions to improve medical school orientations across Canada.

**Instructional Methods:** After a brief introduction to the findings of our research study, an interactive Interview Matrix activity will be facilitated. An Interview Matrix is a tool used to build dialogue and consensus in small groups. Participants will be divided into groups of 4 and invited to discuss predetermined questions with a focus on improving medical school orientation. Following this activity, the entire group will come together to build consensus on the discussed themes in order to develop tangible solutions that can be proposed to Canadian medical schools and administrators.

**Target audience:** Current medical students, recent graduates, Faculty and administrators interested in improving the experiences of first year medical students.

**Learning Objective:** At the end of the workshop, attendees will be able to:

1. Discuss the barriers and facilitators of effective transition into medical school.
2. Develop tangible strategies and possible solutions to improve medical school orientation.

### WE-3 Best Practices in CPD - Creating Effective Scientific Planning Committees

**Rationale/Background:** When planning continuing professional development (CPD) activities, the importance of having an effective Scientific Planning Committee (SPC) is paramount (1-2). The need for planning committees with superb skills and knowledge capacities is essential to a highly functional CPD system. Guidelines about the roles and best practices of SPC are rare in the Canadian context (3) but necessary in order to meet the needs of health professionals lifelong learning. The goal of this collaborative and interactive workshop is to discuss a step-wise approach prospective CPD developers can take to ensure a SPC comes together to deliver relevant and effective CPD opportunities while meeting national accreditation standards.

**Instructional Methods:** We will use a combination of brief didactic sessions by the three presenters with multiple active learning techniques which will form the majority of the workshop. Active learning techniques will include web-enabled audience participation techniques (Sli-do), table exercises, and case-based facilitated discussions. Tools and resources for effective SPC practices will be shared. Participants are welcome to bring CPD program ideas for discussion.

**Target audience:** CPD developers, leaders, program/conference directors. Those interested in developing a CPD educational opportunity and would benefit from a step-wise approach for effective Scientific Planning Committee practices.

**Learning Objective:**

1. Describe the essential roles of effective Scientific Planning Committee members in the planning, development, and delivery of CPD activities
2. Detail an agenda for an effective initial Scientific Planning Committee meeting
3. Apply the ethical principles of the National Standard for Support of Accredited CPD activities as well as the Canadian Medical Association Guidelines for Physician Interaction with Industry (2007)

### WE-4 How to Design a CBME Program Evaluation Plan: Program Theory and Logic Models

**Rationale/Background:** A key element of managing change in education is evaluating new innovations, such as the new training model called Competency-Based Medical Education (CBME). In this workshop, we will demonstrate how the use of an Original Program Theory acts as a framework in the development of a logic model and promotes continuous quality improvement. The session will include information on how to develop an Original Program Theory; how to use program theory to construct a logic model with short, medium, and long-term outcomes; how to set goals for evaluation that consider resources; and strategies for creating an Updated Program Theory. The purpose of this workshop is twofold: First, participants will learn how a program theory can be used as a flexible framework as they work toward drafting an evaluation plan. Second, participants will experience the utility of a program theory as they develop their logic model and overall evaluation strategy.

**Instructional Methods:** This interactive workshop is designed to engage participants to think about complex program evaluation. Through table work and large group discussions, participants will explore what they may wish to include in their own evaluation plan.

**Target audience:** Individuals participating in the evaluation of CBME implementation processes and outcomes.

**Learning Objective:** By the end of the workshop, participants will be able to:

1) Create an original program theory;
2) Illustrate their program theory through a logic model;
3) Create an evaluation strategy that will inform the evolution of the original program theory by updating with knowledge gained from a continuous quality improvement approach.

### WE-5 Designing Curricula to Maximize Learning

**Rationale/Background:** Study methods used by many students in undergraduate degree programs are typically ineffective in medicine. We can influence knowledge retention and recall using different teaching strategies. Research has shown that spaced learning, interleaving and testing are effective ways in which to enhance knowledge retention. Cognitive load is an important consideration when teaching complex key concepts. This workshop explores how we can incorporate our understanding of knowledge retention and recall to enhance learning in our medical curricula, and encourage effective study strategies in our learners.

**Instructional Methods:** The format includes brief interactive presentations interspersed with individual and group activities. Participants will engage with known research around knowledge retention and recall and apply this to their own teaching experience. The techniques used in the workshop will demonstrate the different methods participants can incorporate into their own teaching, and in turn, encourage their learners to adopt. Specific activities: 1. Consider the methods they used to study during training and place in sequence the success rate of different study strategies currently used by students 2. Think, pair share around their own experiences of learning and the strategies used 3. Identify a teaching activity in their own institution and consider how they might change the teaching format to maximize comprehension, retention and recall.

**Target audience:** Educators with an interest in designing curricula that maximize comprehension, retention and recall of knowledge.

**Learning Objective:** By the end of this workshop, participants will be able to:

1. Explain why the more common study methods employed by students are ineffective
2. Give examples of teaching strategies that maximize learning and recall
3. Contrast current curricular teaching methods with proven strategies to maximize learning
4. Apply the concept of scaffolding to a course or topic they are responsible for teaching

### WE-6 Fundraising 101 for Medical Educators

**Rationale/Background:** This is a time of economic and political challenges in higher education. Universities face increasing threats to their business models; they rely more than ever on fundraising dollars. The purpose of this workshop is to raise awareness about Fundraising role in medical education and to reflect on strategies that can be used to ensure successful application of fundraising in an academic environment.

**Instructional Methods:** This interactive workshop will include three parts: • Part one: Fundraising experts will give short presentations on basic fundraising principals in higher education and how these can be used and benefit medical educators. • Part two:

Attendees will be divided into small groups to work on real-life fundraising cases in Education, Research, and Clinical setting. The Fundraising experts will be circulating and facilitating the group discussions. • Part Three: Each group will present their Fundraising solutions and ideas for general consideration.

**Target audience:** Medical educators, clinicians, leaders, faculty administrators, and students

**Learning Objective:** Upon completion of this session, attendees will be able to:

- Define the basics of fundraising and the role of medical educators in forming relationships with potential donors and past alumni.
- Discuss philanthropic trends in higher education.
- Explain the role of AI in fundraising, pipeline analytics, and wealth assessment

### WE-7 Providing Care for LGBTQ+ Patients: Healthcare Provider Allyship Workshop

**Rationale/Background:** Lesbian, gay, bisexual, and transgender (LGBTQ+) individuals are medically underserved populations who face health inequities. Negative encounters with healthcare providers contribute to avoidance of care by LGBTQ+ patients. Research by one of the co-presenters (MS) found that LGBTQ+-identified patients value healthcare providers who are knowledgeable, provide supportive actions, avoid assumptions, and emphasize culturally affirming care. Allyship refers to taking action on behalf of an underserved group, despite not necessarily identifying as part of that group. This workshop provides attendees with a solutions-focused framework to become allies for their LGBTQ+ patients.

**Instructional Methods:** The workshop begins with a brief introduction to key terminology and medically relevant historical information regarding LGBTQ+ health disparities. For the remainder of the workshop, clinical scenarios addressing various aspects of LGBTQ+ care will be discussed with the facilitators. These scenarios are designed to highlight opportunities for allyship. Attendees will be challenged to identify and address their own biases and assumptions, and learn about concrete actions they can take to provide patient-centred care for their LGBTQ+ patients. After discussion of the clinical scenarios, videos showing example clinical encounters (with standardized patient actors) will be shown to illustrate concepts and skills covered during the workshop.

**Target audience:** Health care professionals and trainees (at all levels) who care for LGBTQ+-identified patients.

**Learning Objective:**

1. Describe the systemic health inequities faced by LGBTQ+ patients
2. Define common terminology used by the LGBTQ+ community for self-identification
3. Discuss practical tips for providing safe and respectful care to LGBTQ+ patients.

### Tuesday, April 21st - 08:30-10:00

## Workshop Presentation – Block - F

### WF-1 Responding to the Opioid Crisis by Designing and Developing a National UGME Competency-Based Curriculum on Pain Medicine, Addictions and Substance Use Disorder in Canada

**Rationale/Background:** In 2018, the Association of Faculties of Medicine of Canada undertook a substantive initiative, supported by Health Canada, originally titled "Academic Medicine Responds to the Opioid Crisis: Developing a Canada wide, competency-based online curriculum for future physicians in pain management, substance abuse and addictions". This workshop provides participants with an approach for addressing the largest current public health crisis in Canada by creating an educational opportunity targeting undergraduate medical students to gain knowledge, confidence, competence and motivation to diagnose, treat and manage pain with an understanding of addictions, substance use disorder and harm reduction, while supporting the social accountability mandates of medical schools.

**Instructional Methods:** This workshop will begin with an introduction to the development of the curriculum, then a demonstration of the design elements used such as the spiral curriculum and adoption of formative and synoptic assessments. Participants will engage in an interactive large group discussion on the strengths and challenges of instructional design that promotes national collaboration and a standardized educational program.

**Target audience:** This session is best suited for medical educators and students who have an interest in teaching methods related to pain management, substance use, harm reduction, and/or curriculum development.

**Learning Objective:** At the end of this workshop, you will be able to:

1. Describe how AFMC's Response to Opioids Project can impact the largest public health crisis in Canada.
2. Identify the challenges and opportunities in building a national competency-based online bilingual curriculum for undergraduate medical learners.
3. Explore high-impact practices for engaging health and education stakeholders to support consensus building.

### WF-2 Managing Academic Underperformance: A CMPA Perspective

**Rationale/Background:** Program Directors and other faculty members have the complex task of enabling the achievement of milestones for their residents while also contributing to the development of a positive learning culture. Developing a framework for managing both individual performance and system improvements can benefit both residents and faculty. This instructor-led, participant-centered workshop will introduce participants to the attitudes, knowledge, and skills needed to build a workplace culture that promotes and prioritizes safe medical care and creates accountability in learners and their faculty. Participants will discuss the role of behavioural drift in the development of underperformance. Using their lived experiences to provide examples of each, participants will distinguish between human error, at-risk behaviour and reckless behaviour and, using an algorithmic approach, learn how to leverage institutional values to manage each situation appropriately.

**Instructional Methods:** This workshop uses an instructor-led, participant-centred design where, after a brief introduction, participants collaboratively use a workbook-based case to identify the issues to be discussed next. Participants will share their insights through discussion and voting. They will then be presented with an algorithm and be invited to use it to re-work the case, gaining new insights into its management. Brief revisit and commitment to change exercises end the session.

**Target audience:** Academic Program Directors and program eads as well as preceptors of medical students, residents and fellows.

**Learning Objective:**

1. Describe the three behaviours that threaten patient safety
2. Describe the appropriate management intervention for each behaviour
3. Name the elements of accountability at the system and provider levels

### WF-3 Supporting Wellness through Reflection and Dialogue

**Rationale/Background:** The Royal College challenges educators to prepare physicians who are able to balance personal and professional priorities, incorporate self care, and develop personal and professional awareness and insight. This workshop will present our approach to supporting trainee wellness through reflection and dialogue though a longitudinal curriculum. Students engage with residents and faculty and the formal curriculum during scheduled group meetings over 4 years. Participants are provided with a description of the thematic session and companion pieces, and come prepared to describe an experience and their reflection on it. Students are also required to meet with faculty twice yearly for progress review meetings, and wellness is a theme that often emerges. The informal curriculum, of relationship building, is perhaps the most effective component of the curriculum.

**Instructional Methods:** Our approach to supporting wellness through a Portfolio curriculum will be described through a didactic presentation. Participants will then have the opportunity to simulate a small reflective practice group. Following a demonstration, they will also engage in a coaching conversation around wellness. Participants will discuss how this approach might be adapted to their own education context.

**Target audience:** Medical educators and trainees at all levels

**Learning Objective:** As a result of attending this workshop, participants will be able to:

1. Describe the key components of a curriculum that supports student wellness
2. Participate in/facilitate a small reflective practice group and coaching conversation
3. Leave with the ability to implement a similar curriculum within their own program

### WF-4 Managing Mentorship from the Middle

**Rationale/Background:** Mentorship in health professions education is a well-recognized process for promoting individualized career development and support, and enhancing workplace satisfaction and engagement. Early-to-mid career academic clinicians often find themselves in a position of being tasked to provide mentorship to trainees and junior colleagues. However, these educators may simultaneously be engaged in the process of searching for their own mentoring relationships to address needs related to evolving career and leadership roles. Benefits of mentoring have been described for both the mentor and mentee, but any successful relationship requires connection, commitment and purposeful activity from both participants. In this workshop, participants will review key tasks for successful mentoring from both the mentor and mentee perspectives.

**Instructional Methods:** Drawing on content from both the medical education and business literature, this workshop will utilize reflection and case-based discussion to enable participants to review key tasks for successful mentoring. Interactive activities will include an arts-based icebreaker activity, think-pair-share and large group discussions, a self-assessment checklist for identifying personal mentorship gaps/needs, and a peer-coaching activity for development of action plans to enact future goals. Participants will be encouraged to share and learn from each other's experience, with practical guidance provided by workshop facilitators experienced in faculty development and academic mentorship.

**Target audience:** Early-to-mid career medical educators, but all are welcome.

**Learning Objective:** By the end of this workshop, participants will be able to:

1. Describe key tasks of the mentor and the mentee in an effective mentoring relationship
2. Reflect on past mentorship experiences and identify current mentorship needs / gaps
3. List strategies for acquiring new mentorship for oneself

### WF-5 Enhancing authenticity in SP based OSCE stations: Harnessing insights from the performing arts

**Rationale/Background:** Forms of behavioural assessment, such as the Objective Structured Clinical Examinations (OSCEs), are widely used in health profession education. In their summative form, they strive to facilitate valid and reliable measurements of behavioural competency skills. Many OSCE stations are socially situated activities, where candidates interact with a 'patient' (e.g. a simulated participant SP). Concerns exist about how such constructed forms of assessment fall short of reflecting clinical practice. If assessment does drive learning, are OSCEs drive test performance rather than clinical performance? How can authenticity be improved, whilst retaining reliability?

**Instructional Methods:** • General introductions, 'ground rules' and overview of session. • Introduction to assessment of clinical competency, with focus on the OSCE and characteristics of 'best practice' in assessment. • Provide an overview of the 'lifecycle' of developing an OSCE station. • Buzz group activity regarding difficult SP OSCE stations to write. • 'Think, pair and share' activity regarding simulation /performing arts based techniques and technologies to assist in delivering authentic SP OSCE stations. Some examples with be demonstrated for an immersive experience. • Conclusion, wrap up and take forward messages.

**Target audience:** Health professional researchers and educators who design, deliver and implement OSCEs and anyone who is keen to develop more authentic SP based OSCE stations.

**Learning Objective:**

1) Describe the 'life cycle' of developing an OSCE station.
2) Offer techniques, grounded in the disciplines of simulation, and the performing arts, that can enhance authenticity in SP based OSCE stations - including:
    a. Role development
    b. Script development (including real patient engagement)
    c. 'Props' and 'customs'
    d. Scenography
3) Promote critical reflection of how best to translate this knowledge into educational practice whilst sufficient levels of reliability.

### WF-6 May the Best Team Win - Escape Rooms, Patient Safety and Interprofessional Collaboration

**Rationale/Background:** Serious games have been described in health professional education, incorporating games with specific learning outcomes, structured debrief and reflection as part of the game experience2. Interprofessional collaboration (IPC) competencies are key to practice in a simulated environment but can be challenging to operationalize at a sustainable scale. This workshop aims to explore IPC competencies via participation in a patient-safety oriented escape room - implemented as a serious game.

**Instructional Methods:** After a brief review of serious games and interprofessional collaboration competencies, participants will complete a 30-minute patient safety-oriented escape room, followed by a debrief and reflection on participants' individual and collective experiences in completing the escape room. Participants will review IPC competencies in this context. The workshop will conclude with action-oriented discussion about use of this method for teaching IPC, how to target this type of session across the educational continuum and tips on puzzle development.

**Target audience:** Learners, clinicians and educators from across the educational continuum and health professions.

**Learning Objective:**

1. Practice collaborative and communication skills within a team while attempting to successfully complete the simulated patient safety escape room
2. Reflect on own and team experience in participating in simulated patient safety escape room environment and application of IPC competencies
3. Discuss use of this technique in providing educational opportunities in IPC

### WF-7 Using Systems-Based CPD to Build Mental Health Capacity in Underserved Areas

**Rationale/Background:** Rural and remote regions tend to have higher incidences of mental health disorders, with much of this care being managed within primary care; however, primary care teams have limited training and access to psychiatric support. Traditional Telepsychiatry provides an option to increase access to care; however, when offered in isolation does not focus on building skills and competence for providers to allow them to provide care to their communities. Innovative capacity building approaches where CPD is rooted in the workplace and integrated with specific care delivery models are needed. This workshop will describe the Telepsychiatry Integrated Care Model and Project ECHO to demonstrate a systems-based approach to CPD for mental health. It will explore an approach to developing and implementing CPD integrated within a broader system and how it can be leveraged to build capacity.

**Instructional Methods:** This session engages participants through didactic presentation, and interactive large group and small table discussions. It includes the following: • Introduction to mental health challenge in Canada • Facilitated group discussion: Participant's experiences with capacity building/CPD programs • Overview of the Integrated Care Model and Project ECHO • Small table discussion: Applicability of the models to participants own settings • Final wrap up discussion and brainstorm for next steps

**Target audience:** Individuals interested in learning about capacity building outreach models to improve mental health access, CPD, Faculty Development

**Learning Objective:** Participants will be able to:

- Describe an approach to delivering CPD within outreach models
- Identify frameworks in health professions education that can be utilized for technology
- Enhanced capacity building in rural and underserved communities

### WF-8 National implementation and local differences: The Canadian family medicine residency experience ten years after introducing competency-based medical education

**Overview:** When the College of Family Physicians of Canada (CFPC) began planning for a transition to CBME, the importance of local context was recognized from the earliest stages. The CFPC made the decision to approach CBME as a collaborative process, where high level guidelines would be established by the CFPC (and included in accreditation requirements), but built adequate flexibility into those guidelines to allow individual programs to develop and implement tools and processes that worked for their unique local context. The result of this strategy was the implementation of the Triple C Competency-Based curriculum (Triple C) across all 17 programs between 2009 and 2015. Evaluation data of the Triple C at national and at local levels indicates implementation was successful on many outcome measures. The presentations and discussion will highlight and give voice to diverse strategies and innovations, and will offer ideas and reassurance to educators at all levels who are facing system changes in adopting competency-based assessment. The discussant will summarize the differences between programs while highlighting the alignment with high level guidelines, before opening the floor to questions from the audience.

### WF-9 Matchmaking: Designing and Implementing a Novel Curriculum to Improve Preparation of Medical Students for the Residency Match

**Overview:** A successful residency match could be viewed as having two outcomes of equal importance: choosing a discipline and then matching to that discipline. We propose a novel curriculum addressing both steps.

JOURNAL INFORMATION
==============================
NLM Title Abbreviation: Can Med Educ J
Journal ID: Can Med Educ J
NLM Journal ID: 101560935
Journal ID: CMEJ

Canadian Medical Education Journal

EISSN: 1923-1202
Publisher: University of Saskatchewan

ARTICLE INFORMATION
==============================
Subjects: Wa-1

Successful “in-the-moment” feedback and coaching conversations in the era of CBME and workplace-based assessment

Heather Armson University of Calgary

Jocelyn Lockyer University of Calgary

Joan Sargeant Dalhousie University

Marygrace Zetkulic Hackensack University

Amanda Roze des Ordons University of Calgary

Jessica Trier Queen’s University

Karen Konings Maastricht University

Subha Ramani Harvard University

Electronic publication date: 2020 Apr 18

JOURNAL INFORMATION
==============================
NLM Title Abbreviation: Can Med Educ J
Journal ID: Can Med Educ J
NLM Journal ID: 101560935
Journal ID: CMEJ

Canadian Medical Education Journal

EISSN: 1923-1202
Publisher: University of Saskatchewan

ARTICLE INFORMATION
==============================
Subjects: Wa-2

Navigating the Evidence Synthesis Landscape: Exploring Fundamentals of common evidence synthesis approaches in medical education

Tanya Horsley The Royal College of Physicians and Surgeons

Ryan Brydges University of Toronto

Rachel Ellaway University of Calgary

Electronic publication date: 2020 Apr 18

JOURNAL INFORMATION
==============================
NLM Title Abbreviation: Can Med Educ J
Journal ID: Can Med Educ J
NLM Journal ID: 101560935
Journal ID: CMEJ

Canadian Medical Education Journal

EISSN: 1923-1202
Publisher: University of Saskatchewan

ARTICLE INFORMATION
==============================
Subjects: Wa-3

Strengthening Health Equity in Undergraduate Medical Education and Continuing Professional Development: Using storytelling and role play to build competencies

Lloy Wylie Western University

Electronic publication date: 2020 Apr 18

JOURNAL INFORMATION
==============================
NLM Title Abbreviation: Can Med Educ J
Journal ID: Can Med Educ J
NLM Journal ID: 101560935
Journal ID: CMEJ

Canadian Medical Education Journal

EISSN: 1923-1202
Publisher: University of Saskatchewan

ARTICLE INFORMATION
==============================
Subjects: Wa-4

Leveraging Data Visualization and Data Storytelling in Programmatic Assessment and Program Evaluation

Yuxin Tu University of Toronto

Pauline Pan University of Toronto

Richard Pittini University of Toronto

Electronic publication date: 2020 Apr 18

JOURNAL INFORMATION
==============================
NLM Title Abbreviation: Can Med Educ J
Journal ID: Can Med Educ J
NLM Journal ID: 101560935
Journal ID: CMEJ

Canadian Medical Education Journal

EISSN: 1923-1202
Publisher: University of Saskatchewan

ARTICLE INFORMATION
==============================
Subjects: Wa-5

Getting your ducks in a line: enhancing research congruence

Helen Reid Queen's University, Belfast

Richard Conn Queen's University Belfast

Grainne Kearney Queen's University Belfast

Gerry Gormley Queen's University Belfast

Electronic publication date: 2020 Apr 18

JOURNAL INFORMATION
==============================
NLM Title Abbreviation: Can Med Educ J
Journal ID: Can Med Educ J
NLM Journal ID: 101560935
Journal ID: CMEJ

Canadian Medical Education Journal

EISSN: 1923-1202
Publisher: University of Saskatchewan

ARTICLE INFORMATION
==============================
Subjects: Wa-6

Price of a dream: Strategies to reduce medical school application costs for students from low socioeconomic status backgrounds

Ike Okafor University of Toronto

Justin Lam University of Toronto

Chantal Phillips University of Toronto

Maisoon D. Yousif Dalhousie University

Electronic publication date: 2020 Apr 18

JOURNAL INFORMATION
==============================
NLM Title Abbreviation: Can Med Educ J
Journal ID: Can Med Educ J
NLM Journal ID: 101560935
Journal ID: CMEJ

Canadian Medical Education Journal

EISSN: 1923-1202
Publisher: University of Saskatchewan

ARTICLE INFORMATION
==============================
Subjects: Wa-7

Reviewing in Medical Education: The CMEJ experience

Marcel D'Eon University of Saskatchewan

Electronic publication date: 2020 Apr 18

JOURNAL INFORMATION
==============================
NLM Title Abbreviation: Can Med Educ J
Journal ID: Can Med Educ J
NLM Journal ID: 101560935
Journal ID: CMEJ

Canadian Medical Education Journal

EISSN: 1923-1202
Publisher: University of Saskatchewan

ARTICLE INFORMATION
==============================
Subjects: Wb-2

Intégrer des patients partenaires: comprendre les enjeux terrains

Marie-Pierre Codsi Université de Montréal

Philippe Karazivan Université de Montréal

Annie Descôteaux Université de Montréal

Mathieu Jackson Université de Montréal

Clara Dallaire Université de Montréal

Louise Nicaise Université de Montréal

Antoine Boivin Université de Montréal

Electronic publication date: 2020 Apr 18

JOURNAL INFORMATION
==============================
NLM Title Abbreviation: Can Med Educ J
Journal ID: Can Med Educ J
NLM Journal ID: 101560935
Journal ID: CMEJ

Canadian Medical Education Journal

EISSN: 1923-1202
Publisher: University of Saskatchewan

ARTICLE INFORMATION
==============================
Subjects: Wb-3

Peer observation of teaching: the next step in Faculty Development

Lyne Menard Université de Montréal

Sophie Galarneau Université de Montréal

Alain Papineau Université de Montréal

Electronic publication date: 2020 Apr 18

JOURNAL INFORMATION
==============================
NLM Title Abbreviation: Can Med Educ J
Journal ID: Can Med Educ J
NLM Journal ID: 101560935
Journal ID: CMEJ

Canadian Medical Education Journal

EISSN: 1923-1202
Publisher: University of Saskatchewan

ARTICLE INFORMATION
==============================
Subjects: Wb-4

Professionalism: Developing a Vision for Evidence Informed Curriculum

Sue Murphy University of British Columbia

Susan Paul University of British Columbia

Janet Lundie University of British Columbia

Electronic publication date: 2020 Apr 18

JOURNAL INFORMATION
==============================
NLM Title Abbreviation: Can Med Educ J
Journal ID: Can Med Educ J
NLM Journal ID: 101560935
Journal ID: CMEJ

Canadian Medical Education Journal

EISSN: 1923-1202
Publisher: University of Saskatchewan

ARTICLE INFORMATION
==============================
Subjects: Wb-6

Making workplace-based assessment work; recognizing opportunities and limitations

Pim Teunissen Maastricht University, School of Health Professions Education (SHE)

Erik Driessen Maastricht University, School of Health Professions Education (SHE)

Electronic publication date: 2020 Apr 18

JOURNAL INFORMATION
==============================
NLM Title Abbreviation: Can Med Educ J
Journal ID: Can Med Educ J
NLM Journal ID: 101560935
Journal ID: CMEJ

Canadian Medical Education Journal

EISSN: 1923-1202
Publisher: University of Saskatchewan

ARTICLE INFORMATION
==============================
Subjects: Wb-7

Applying the 3-P Model to Plan and Evaluate your Interprofessional Education Activity

Marcio Gomes University of Ottawa

Denyse Richardson University of Toronto

Jerry Maniate University of Ottawa

Samantha Halman University of Ottawa

Linda Snell McGill

Electronic publication date: 2020 Apr 18

JOURNAL INFORMATION
==============================
NLM Title Abbreviation: Can Med Educ J
Journal ID: Can Med Educ J
NLM Journal ID: 101560935
Journal ID: CMEJ

Canadian Medical Education Journal

EISSN: 1923-1202
Publisher: University of Saskatchewan

ARTICLE INFORMATION
==============================
Subjects: Wc-2

Practical Strategies for Meaningful Allyship in Indigenous Health and Beyond

Ming-Ka Chan University of Manitoba

Javeed Sukhera Western University

Lisa Richardson University of Toronto

Jerry Maniate University of Ottawa

Adrienne Morrow University of Manitoba

Marcia Anderson DeCoteau University of Manitoba

Electronic publication date: 2020 Apr 18

JOURNAL INFORMATION
==============================
NLM Title Abbreviation: Can Med Educ J
Journal ID: Can Med Educ J
NLM Journal ID: 101560935
Journal ID: CMEJ

Canadian Medical Education Journal

EISSN: 1923-1202
Publisher: University of Saskatchewan

ARTICLE INFORMATION
==============================
Subjects: Wc-3

Teaching how to deal with emotions during difficult conversations: Opportunities and challenges

Anne Kawamura University of Toronto

Dominique Piquette University of Toronto

Briseida Mema University of Toronto

Electronic publication date: 2020 Apr 18

JOURNAL INFORMATION
==============================
NLM Title Abbreviation: Can Med Educ J
Journal ID: Can Med Educ J
NLM Journal ID: 101560935
Journal ID: CMEJ

Canadian Medical Education Journal

EISSN: 1923-1202
Publisher: University of Saskatchewan

ARTICLE INFORMATION
==============================
Subjects: Wc-4

Discovering Cultural Humility in Healthcare Education

Dalia Al Mouaswas University Health Netwoek

Mohammad Salhia University Health Network

Electronic publication date: 2020 Apr 18

JOURNAL INFORMATION
==============================
NLM Title Abbreviation: Can Med Educ J
Journal ID: Can Med Educ J
NLM Journal ID: 101560935
Journal ID: CMEJ

Canadian Medical Education Journal

EISSN: 1923-1202
Publisher: University of Saskatchewan

ARTICLE INFORMATION
==============================
Subjects: Wc-5

Side by Side : A peer support program for medical students at the University of Ottawa

Kay-Anne Haykal University of Ottawa

Kelsey Mongrain University of Ottawa

Electronic publication date: 2020 Apr 18

JOURNAL INFORMATION
==============================
NLM Title Abbreviation: Can Med Educ J
Journal ID: Can Med Educ J
NLM Journal ID: 101560935
Journal ID: CMEJ

Canadian Medical Education Journal

EISSN: 1923-1202
Publisher: University of Saskatchewan

ARTICLE INFORMATION
==============================
Subjects: Wc-6

Deriving Logic in Program Evaluation: Application of Logic Model Frameworks in Health Professions Education

David Rojas University of Toronto

Betty Onyura University of Toronto

Theresa Beesley McGill

Tanya Horsley The Royal College of Physicians and Surgeons

Electronic publication date: 2020 Apr 18

JOURNAL INFORMATION
==============================
NLM Title Abbreviation: Can Med Educ J
Journal ID: Can Med Educ J
NLM Journal ID: 101560935
Journal ID: CMEJ

Canadian Medical Education Journal

EISSN: 1923-1202
Publisher: University of Saskatchewan

ARTICLE INFORMATION
==============================
Subjects: Wc-7

Capacity Building for Case-based Quality and Safety Education using QuaCC: the Quality Care Curriculum

Siobhán Neville University of Toronto

Brie Yama University of Toronto

Julie Johnstone University of Toronto

Zia Bismilla University of Toronto

Electronic publication date: 2020 Apr 18

JOURNAL INFORMATION
==============================
NLM Title Abbreviation: Can Med Educ J
Journal ID: Can Med Educ J
NLM Journal ID: 101560935
Journal ID: CMEJ

Canadian Medical Education Journal

EISSN: 1923-1202
Publisher: University of Saskatchewan

ARTICLE INFORMATION
==============================
Subjects: Wd-1

Assessment of the Individual within a Team Context

Briseida Mema University of Toronto

Anne Kawamura University of Toronto

Dominique Piquette University of Toronto

Electronic publication date: 2020 Apr 18

JOURNAL INFORMATION
==============================
NLM Title Abbreviation: Can Med Educ J
Journal ID: Can Med Educ J
NLM Journal ID: 101560935
Journal ID: CMEJ

Canadian Medical Education Journal

EISSN: 1923-1202
Publisher: University of Saskatchewan

ARTICLE INFORMATION
==============================
Subjects: Wd-7

Coping with Imposter Syndrome in Medical Training

Anita Gupta University of Toronto

Martha Chamodraka McGill

Jaylin Bradbury University of Toronto

Electronic publication date: 2020 Apr 18

JOURNAL INFORMATION
==============================
NLM Title Abbreviation: Can Med Educ J
Journal ID: Can Med Educ J
NLM Journal ID: 101560935
Journal ID: CMEJ

Canadian Medical Education Journal

EISSN: 1923-1202
Publisher: University of Saskatchewan

ARTICLE INFORMATION
==============================
Subjects: Wd-6

Raising critical consciousness: introducing social justice to generalist clinical teaching

Jenny Johnston Queen’s University Belfast

Annalisa Manca Queen’s University Belfast

Lindsey Pope Glasgow University

Helen Reid Queen’s University Belfast

Cynthia Whitehead University of Toronto

Electronic publication date: 2020 Apr 18

JOURNAL INFORMATION
==============================
NLM Title Abbreviation: Can Med Educ J
Journal ID: Can Med Educ J
NLM Journal ID: 101560935
Journal ID: CMEJ

Canadian Medical Education Journal

EISSN: 1923-1202
Publisher: University of Saskatchewan

ARTICLE INFORMATION
==============================
Subjects: Wd-5

“Tuning in to Collaborative Healthcare: a new interprofessional education experience in the performing arts.”

Willem Blois Dalhousie University

Julia LeBlanc University of Ottawa

Electronic publication date: 2020 Apr 18

JOURNAL INFORMATION
==============================
NLM Title Abbreviation: Can Med Educ J
Journal ID: Can Med Educ J
NLM Journal ID: 101560935
Journal ID: CMEJ

Canadian Medical Education Journal

EISSN: 1923-1202
Publisher: University of Saskatchewan

ARTICLE INFORMATION
==============================
Subjects: Wd-4

Is conducting a BEME review worth it? Advantages, challenges, and practical tips

Sara MortazHejri McGill

Yvonne Steinert McGill

Electronic publication date: 2020 Apr 18

JOURNAL INFORMATION
==============================
NLM Title Abbreviation: Can Med Educ J
Journal ID: Can Med Educ J
NLM Journal ID: 101560935
Journal ID: CMEJ

Canadian Medical Education Journal

EISSN: 1923-1202
Publisher: University of Saskatchewan

ARTICLE INFORMATION
==============================
Subjects: Wd-3

Educating future educators: Designing and implementing elective courses in medical and health sciences education

Stuart Lubarsky McGill

Monika Kvernenes University of Bergen

Robert Sternszus McGill

Edvin Schei University of Bergen

Electronic publication date: 2020 Apr 18

JOURNAL INFORMATION
==============================
NLM Title Abbreviation: Can Med Educ J
Journal ID: Can Med Educ J
NLM Journal ID: 101560935
Journal ID: CMEJ

Canadian Medical Education Journal

EISSN: 1923-1202
Publisher: University of Saskatchewan

ARTICLE INFORMATION
==============================
Subjects: Wd-2

Medical student mistreatment 2020: The Calgary Experience of Pushing for Culture Shift Using Faculty Advocates. A Case-based Workshop

Sarah Weeks University of Calgary

Deirdre Jenkins University of Calgary

Yves Starreveld University of Calgary

Kevin Busche University of Calgary

Ellen McLeod University of Calgary

Sylvain Coderre University of Calgary

Electronic publication date: 2020 Apr 18

JOURNAL INFORMATION
==============================
NLM Title Abbreviation: Can Med Educ J
Journal ID: Can Med Educ J
NLM Journal ID: 101560935
Journal ID: CMEJ

Canadian Medical Education Journal

EISSN: 1923-1202
Publisher: University of Saskatchewan

ARTICLE INFORMATION
==============================
Subjects: Wc-1

Building Gryffindor: Revitalizing the Clinical Education Environment for Engagement & Learning

Christie Newton University of British Columbia

Jacqueline Ashby University of British Columbia

Electronic publication date: 2020 Apr 18

JOURNAL INFORMATION
==============================
NLM Title Abbreviation: Can Med Educ J
Journal ID: Can Med Educ J
NLM Journal ID: 101560935
Journal ID: CMEJ

Canadian Medical Education Journal

EISSN: 1923-1202
Publisher: University of Saskatchewan

ARTICLE INFORMATION
==============================
Subjects: We-1

Serious about Gaming: A workshop for budding serious board game developers

Teresa Chan McMaster University

Clare Wallner McMaster University

Jasmine Liu McMaster University

Sarrah Lal McMaster University

Chad Singh McMaster University

Sonja Wakeling McMaster University

Anuja Bhalerao University of Toronto

Electronic publication date: 2020 Apr 18

JOURNAL INFORMATION
==============================
NLM Title Abbreviation: Can Med Educ J
Journal ID: Can Med Educ J
NLM Journal ID: 101560935
Journal ID: CMEJ

Canadian Medical Education Journal

EISSN: 1923-1202
Publisher: University of Saskatchewan

ARTICLE INFORMATION
==============================
Subjects: We-8

Teaching the Patient's Medical Home - from policy to practice

Martina Kelly University of Calgary

Amanda Condon University of Manitoba

Dana Turcotte University of Manitoba

Divya Garg University of Calgary

Electronic publication date: 2020 Apr 18

JOURNAL INFORMATION
==============================
NLM Title Abbreviation: Can Med Educ J
Journal ID: Can Med Educ J
NLM Journal ID: 101560935
Journal ID: CMEJ

Canadian Medical Education Journal

EISSN: 1923-1202
Publisher: University of Saskatchewan

ARTICLE INFORMATION
==============================
Subjects: We-9

Educating for Critical Consciousness in the Clinical Teaching Environment

Lindsay Herzog University of Toronto

Lindsay Baker University of Toronto

Stella Ng University of Toronto

Lisa Richardson University of Toronto

Beck McNeil University of Toronto

Victoria Boyd University of Toronto

Emilia Kangasjarvi University of Toronto

Electronic publication date: 2020 Apr 18

JOURNAL INFORMATION
==============================
NLM Title Abbreviation: Can Med Educ J
Journal ID: Can Med Educ J
NLM Journal ID: 101560935
Journal ID: CMEJ

Canadian Medical Education Journal

EISSN: 1923-1202
Publisher: University of Saskatchewan

ARTICLE INFORMATION
==============================
Subjects: We-2

“Welcome to medical school!”: Designing an inclusive medical school orientation for medical students

Asia van Buuren University of Toronto

Wid Yaseen University of Toronto

Paula Veinot Independent Research Consultant, Halifax, Nova Scotia, Canada

Maria Mylopoulos University of Toronto

Marcus Law University of Toronto

Electronic publication date: 2020 Apr 18

JOURNAL INFORMATION
==============================
NLM Title Abbreviation: Can Med Educ J
Journal ID: Can Med Educ J
NLM Journal ID: 101560935
Journal ID: CMEJ

Canadian Medical Education Journal

EISSN: 1923-1202
Publisher: University of Saskatchewan

ARTICLE INFORMATION
==============================
Subjects: We-3

Best Practices in CPD - Creating Effective Scientific Planning Committees

Suzan Schneeweiss University of Toronto

Janice Harvey The College of Family Physicians of Canada

Valerie Schulz Western University

Electronic publication date: 2020 Apr 18

JOURNAL INFORMATION
==============================
NLM Title Abbreviation: Can Med Educ J
Journal ID: Can Med Educ J
NLM Journal ID: 101560935
Journal ID: CMEJ

Canadian Medical Education Journal

EISSN: 1923-1202
Publisher: University of Saskatchewan

ARTICLE INFORMATION
==============================
Subjects: We-4

How to Design a CBME Program Evaluation Plan: Program Theory and Logic Models

Deena Hamza University of Alberta

Anna Oswald University of Alberta

Electronic publication date: 2020 Apr 18

JOURNAL INFORMATION
==============================
NLM Title Abbreviation: Can Med Educ J
Journal ID: Can Med Educ J
NLM Journal ID: 101560935
Journal ID: CMEJ

Canadian Medical Education Journal

EISSN: 1923-1202
Publisher: University of Saskatchewan

ARTICLE INFORMATION
==============================
Subjects: We-5

Designing Curricula to Maximize Learning

Wendy A Stewart Dalhousie University

Keith Wilson Dalhousie University

Electronic publication date: 2020 Apr 18

JOURNAL INFORMATION
==============================
NLM Title Abbreviation: Can Med Educ J
Journal ID: Can Med Educ J
NLM Journal ID: 101560935
Journal ID: CMEJ

Canadian Medical Education Journal

EISSN: 1923-1202
Publisher: University of Saskatchewan

ARTICLE INFORMATION
==============================
Subjects: We-6

Fundraising 101 for Medical Educators

Alireza Jalali University of Ottawa

Jacline Nyman University of Ottawa

Electronic publication date: 2020 Apr 18

JOURNAL INFORMATION
==============================
NLM Title Abbreviation: Can Med Educ J
Journal ID: Can Med Educ J
NLM Journal ID: 101560935
Journal ID: CMEJ

Canadian Medical Education Journal

EISSN: 1923-1202
Publisher: University of Saskatchewan

ARTICLE INFORMATION
==============================
Subjects: We-7

Providing Care for LGBTQ+ Patients: Healthcare Provider Allyship Workshop

Michael Scott University of Toronto

Tehmina Ahmad University of Toronto

Jeremy Cygler University of Toronto

Wilson Kwong University of Toronto

Miranda Schreiber University of Toronto

Jacqueline James University of Toronto

Electronic publication date: 2020 Apr 18

JOURNAL INFORMATION
==============================
NLM Title Abbreviation: Can Med Educ J
Journal ID: Can Med Educ J
NLM Journal ID: 101560935
Journal ID: CMEJ

Canadian Medical Education Journal

EISSN: 1923-1202
Publisher: University of Saskatchewan

ARTICLE INFORMATION
==============================
Subjects: Wf-1

Responding to the Opioid Crisis by Designing and Developing a National UGME Competency-Based Curriculum on Pain Medicine, Addictions and Substance Use Disorder in Canada

Lisa Graves Western Michigan University Homer Stryker M.D.School of Medicine

Fran Kirby The Association of Faculties of Medicine of Canada

Richard van Wylick Queen’s University

Jeanne Mulder Queen’s University

M.A. Adams Queen’s University

Klodiana Kolomitro Queen’s University

Electronic publication date: 2020 Apr 18

JOURNAL INFORMATION
==============================
NLM Title Abbreviation: Can Med Educ J
Journal ID: Can Med Educ J
NLM Journal ID: 101560935
Journal ID: CMEJ

Canadian Medical Education Journal

EISSN: 1923-1202
Publisher: University of Saskatchewan

ARTICLE INFORMATION
==============================
Subjects: Wf-2

Managing Academic Underperformance: A CMPA Perspective

Steven Bellemare Canadian Medical Protective Association

Guylaine Lefebvre Canadian Medical Protective Association

Electronic publication date: 2020 Apr 18

JOURNAL INFORMATION
==============================
NLM Title Abbreviation: Can Med Educ J
Journal ID: Can Med Educ J
NLM Journal ID: 101560935
Journal ID: CMEJ

Canadian Medical Education Journal

EISSN: 1923-1202
Publisher: University of Saskatchewan

ARTICLE INFORMATION
==============================
Subjects: Wf-3

Supporting Wellness through Reflection and Dialogue

Nirit Bernhard University of Toronto

Susanna Talarico University of Toronto

Susanna Talarico University of Toronto

Electronic publication date: 2020 Apr 18

JOURNAL INFORMATION
==============================
NLM Title Abbreviation: Can Med Educ J
Journal ID: Can Med Educ J
NLM Journal ID: 101560935
Journal ID: CMEJ

Canadian Medical Education Journal

EISSN: 1923-1202
Publisher: University of Saskatchewan

ARTICLE INFORMATION
==============================
Subjects: Wf-4

Managing Mentorship from the Middle

Judith Peranson University of Toronto

Abbas Ghavam-Rassoul University of Toronto

Electronic publication date: 2020 Apr 18

JOURNAL INFORMATION
==============================
NLM Title Abbreviation: Can Med Educ J
Journal ID: Can Med Educ J
NLM Journal ID: 101560935
Journal ID: CMEJ

Canadian Medical Education Journal

EISSN: 1923-1202
Publisher: University of Saskatchewan

ARTICLE INFORMATION
==============================
Subjects: Wf-5

Enhancing authenticity in SP based OSCE stations: Harnessing insights from the performing arts

Gerry Gormley Queen's University Belfast

Helen Reid Queen's University Belfast

Grainne Kearney Queen's University Belfast

Electronic publication date: 2020 Apr 18

JOURNAL INFORMATION
==============================
NLM Title Abbreviation: Can Med Educ J
Journal ID: Can Med Educ J
NLM Journal ID: 101560935
Journal ID: CMEJ

Canadian Medical Education Journal

EISSN: 1923-1202
Publisher: University of Saskatchewan

ARTICLE INFORMATION
==============================
Subjects: Wf-6

May the Best Team Win - Escape Rooms, Patient Safety and Interprofessional Collaboration

Amanda Condon University of Manitoba

Moni Fricke University of Manitoba

Christine Polimeni University of Manitoba

Electronic publication date: 2020 Apr 18

JOURNAL INFORMATION
==============================
NLM Title Abbreviation: Can Med Educ J
Journal ID: Can Med Educ J
NLM Journal ID: 101560935
Journal ID: CMEJ

Canadian Medical Education Journal

EISSN: 1923-1202
Publisher: University of Saskatchewan

ARTICLE INFORMATION
==============================
Subjects: Wf-7

Using Systems-Based CPD to Build Mental Health Capacity in Underserved Areas

Eva Serhal Centre for Addiction and Mental Health

Sanjeev Sockalingam Centre for Addiction and Mental Health

Allison Crawford Centre for Addiction and Mental Health

Cheryl Pereira Centre for Addiction and Mental Health

Jenny Hardy Centre for Addiction and Mental Health

Anne Kirvan Centre for Addiction and Mental Health

Electronic publication date: 2020 Apr 18

JOURNAL INFORMATION
==============================
NLM Title Abbreviation: Can Med Educ J
Journal ID: Can Med Educ J
NLM Journal ID: 101560935
Journal ID: CMEJ

Canadian Medical Education Journal

EISSN: 1923-1202
Publisher: University of Saskatchewan

ARTICLE INFORMATION
==============================
Subjects: Wf-8

National implementation and local differences: The Canadian family medicine residency experience ten years after introducing competency-based medical education

Shelley Ross University of Alberta

Brent Kvern University of Manitoba

Theresa van der Goes University of British Columbia

Luce Pelissier-Simard Université de Sherbrooke

Karen Schultz Queen’s University

Kathy Lawrence University of Saskatchewan

Cheri Bethune Memorial – University of Newfoundland

Kiranpal Dhillon University of Alberta

Electronic publication date: 2020 Apr 18

JOURNAL INFORMATION
==============================
NLM Title Abbreviation: Can Med Educ J
Journal ID: Can Med Educ J
NLM Journal ID: 101560935
Journal ID: CMEJ

Canadian Medical Education Journal

EISSN: 1923-1202
Publisher: University of Saskatchewan

ARTICLE INFORMATION
==============================
Subjects: Wf-9

Matchmaking: Designing and Implementing a Novel Curriculum to Improve Preparation of Medical Students for the Residency Match

Melinda Davis University of Calgary

Kevin McLaughlin University of Calgary

Janeve Desy University of Calgary

Sue-Ann Facchini University of Calgary

Christopher Naugler University of Calgary

Electronic publication date: 2020 Apr 18

